# How is a specialist depression service effective for persistent moderate to severe depressive disorder?: a qualitative study of service user experience

**DOI:** 10.1186/s12888-018-1708-9

**Published:** 2018-06-15

**Authors:** Louise Thomson, Marcus Barker, Catherine Kaylor-Hughes, Anne Garland, Rajini Ramana, Richard Morriss, Emily Hammond, Gail Hopkins, Sandra Simpson

**Affiliations:** 1 Division of Psychiatry and Applied Psychology, Institute of Mental Health, School of Medicine, University of Nottingham, Yang Fujia Building, Nottingham, NG8 1BB UK; 2 CLAHRC-EM, School of Medicine, University of Nottingham, Nottingham, UK; 3 Adult Mental Health Directorate, Nottinghamshire Healthcare Trust, Nottingham, UK; 4 Cambridge and Peterborough Partnership NHS Foundation Trust, Cambridge, UK; 5 Faculty of Science, University of Nottingham, Nottingham, UK; 6 Clinical Research Network, Nottinghamshire Healthcare Trust, Nottingham, UK

**Keywords:** Depression, Cognitive Behavioural therapy, Pharmacological therapy, Service user experience, Qualitative study

## Abstract

**Background:**

A specialist depression service (SDS) offering collaborative pharmacological and cognitive behaviour therapy treatment for persistent depressive disorder showed effectiveness against depression symptoms versus usual community based multidisciplinary care in a randomised controlled trial (RCT) in specialist mental health services in England. However, there is uncertainty concerning how specialist depression services effect such change. The current study aimed to evaluate the factors which may explain the greater effectiveness of SDS compared to Treatment as Usual (TAU) by exploring the experience of the RCT participants.

**Methods:**

Qualitative audiotaped and transcribed semi-structured interviews were conducted 12–18 months after baseline with 21 service users (12 SDS, 9 TAU arms) drawn from all three sites. Inductive thematic analysis using a grounded approach contrasted the experiences of SDS with TAU participants.

**Results:**

Four themes emerged in relation to service user experience: 1. Specific treatment components of the SDS: which included sub-themes of the management of medication change, explaining and developing treatment strategies, setting realistic expectations, and person-centred and holistic approach; 2. Individual qualities of SDS clinicians; 3. Collaborative team context in SDS: which included sub-themes of communication between healthcare professionals, and continuity of team members; 4. Accessibility to SDS: which included sub-themes of flexibility of locations, frequent consultation as reinforcement, gradual pace of treatment, and challenges of returning to usual care.

**Conclusions:**

The study uncovered important mechanisms and contextual factors in the SDS that service users experience as different from TAU, and which may explain the greater effectiveness of the SDS: the technical expertise of the healthcare professionals, personal qualities of clinicians, teamwork, gradual pace of care, accessibility and managing service transitions. Usual care in other specialist mental health services may share many of the features from the SDS.

**Trial registration:**

“Trial of the Clinical and Cost Effectiveness of a Specialist Expert Mood Disorder Team for Refractory Unipolar Depressive Disorder” was registered in www.ClinicalTrials.gov (NCT01047124) on 12–01-2010 and the ISRCTN registry was registered in www.isrctn.com (ISRCTN10963342) on 25–11-2015 (retrospectively registered).

## Background

Major depressive disorder is experienced by up to 15% of people in high income countries at least once in their lifetime [1]. It has been identified as the second leading cause of years lived with disability in the world [2]. Furthermore, recurrence of major depressive disorder is high, rising from a rate of 60% recurrence 5 years after an episode of depression to 85% after 15 years [3].

Combined pharmacotherapy and psychological treatments delivered by specialist multi-professional teams are widely recommended [4]. This collaborative care approach is characterised by joint assessments by psychiatrists and psychological therapists, and the development of structured management plans according to protocols for both psychotherapy and pharmacology based on NICE Guidelines for depression (2009) [5]. However, only a small number of randomised controlled trials (RCTs) have examined the effectiveness of such a collaborative treatment service for persistent, chronic or treatment resistant moderate-to-severe depressive disorder (e.g. [6]). In our primary study, a large scale RCT of a Specialist Depression Service (SDS) providing pharmacotherapy and psychological treatment from a collaborative specialist team [7] showed a significant reduction in depression symptoms after 18 months [8]. However, there is uncertainty about the factors which may contribute to these improved outcomes for people experiencing severe and recurring depression [9] and service users’ experience of these.

There is little research evidence to date examining the factors that influence the effectiveness of collaborative care models for depression. The NICE 2017 draft guidelines for depression [10] includes an analysis showing that improved outcomes from collaborative care for depression were mediated by having stepped care where another treatment is used if the first is not responsive, decision-support between the psychiatrist and psychotherapist, and having a medication algorithm. Longer illness duration is a robust predictor of poor outcome in antidepressant-placebo drug trials and combinations of psychotherapy and antidepressant treatment studies [11–14]. Length of therapeutic intervention appears to be an important factor for longer-term remission from depression symptoms [15], and one trial has found that more than 16 sessions of either CBT or psychodynamic supportive therapy were needed to achieve remission [16]. In psychotherapy research, therapeutic alliance when rated by the patient and adherence by the therapist to the treatment manual have been found to be a moderator of outcomes [17]. Together, these findings suggest the future design of services for people with recurrent depression should aim to foster a longer-term and collaborative approach to building therapeutic relationships.

Qualitative methods have been shown to provide a useful approach to understanding participants’ perceptions and experiences of an intervention and to identify the active ingredients of complex and multi-faceted mental health services [18–21]. Romakkaniemi and Kilpelainen [22] analysed the experiences of two service users with depression through their written blogs and found four themes: a confident working relationship, time and hiatus for finding one’s own authenticity, successful timing of interventions and a holistic view of life. However, this study did not examine the experience of a single service, rather their experience of multiple services over time.

The aims of this qualitative study into service user experiences were to:

- Obtain service user views on their experience of either the SDS or Treatment as Usual (TAU) as part of a large RCT
- Identify the features of the SDS intervention that were experienced as beneficial
- Compare the experience of those in different arms of the study

## Methods

### SDS trial design

The SDS treatment groups received a collaborative care approach between patient, psychiatrist and cognitive behaviour therapist, and, where clinically indicated, contact with the patient’s General Practitioner (GP) and community mental health teams, voluntary sector organisations, family and friends and employers. The SDS collaborative approach began with a joint assessment between service user, psychiatrist and CBT therapist. Follow up sessions using the same format were conducted at 3,6,9 and 12 months. The intervention was characterised by optimised pharmacotherapy (meeting individually with the psychiatrist initially fortnightly tapering to monthly and three monthly once optimised). Alongside this was weekly Beckian CBT with an option for Mindfulness Based Cognitive Therapy as a relapse prevention strategy. From month 10 there was a gradual transition to usual care with either primary care or secondary care community teams after 13 months (range 12–15). The SDS groups also took an active social inclusion and recovery stance [23] and had links with local social inclusion initiatives (i.e. vocational, educational and back to work and self-help support networks). The services aimed to deliver a collaborative and integrated psychobiosocial model of treatment [24] in which equal importance is given to medication and psychosocial interventions as treatments of potential benefit, and the initial treatment rationale presented explicitly articulated the reasons for using each in combination. All treatments were National Institute for Health and Care Excellence (NICE) –recommended [4] and delivered by staff with a high level of training and clinical expertise in treating depression.

TAU was directed by a consultant psychiatrist and consisted of pharmacotherapy, sometimes augmented by psychological interventions but without co-ordination or joint reviews and assessments between healthcare professionals [7, 8].

### Study sites

The Specialised Depression Service was implemented in the three free to the public specialist mental health services operated by the National Health Service in England in Nottinghamshire, Cambridgeshire and Derbyshire.

### Participants in the RCT

**Fig. 1:**
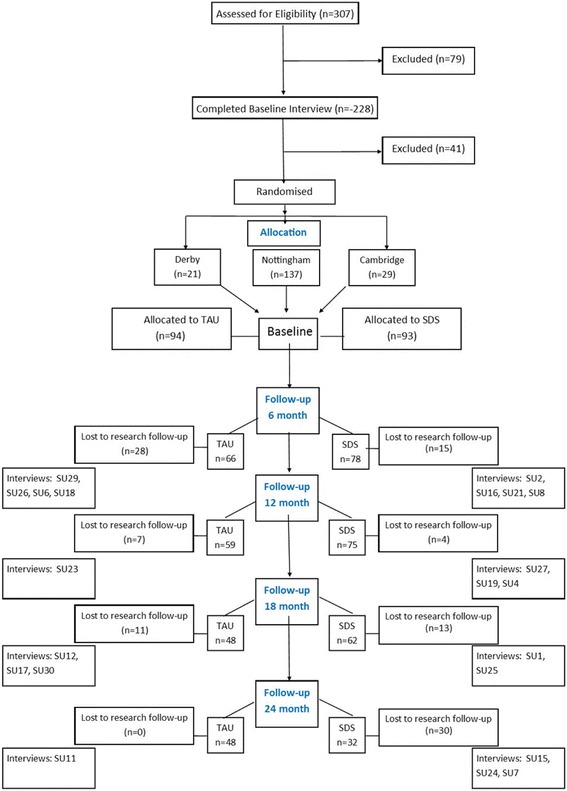
Flow of participants through the trial

The RCT recruited 187 participants with moderate to severe depression from the three sites as follows: Nottingham (137), Derby (21) and Cambridge (29):. 93 were assigned to SDS and 94 to TAU. Figure 1 shows the flow of participants through the trial. Inclusion criteria for the RCT were a primary diagnosis of major depressive disorder; continuing contact with specialist mental health services after 6 months, score 16 or above on the 17-item Hamilton Rating Scale for Depression (HRSD) [25] and 60 or below on the Global Assessment of Functioning (GAF) [26]. Exclusion criteria were such that patients were excluded if they were in receipt of emergency care for suicide risk, homicide risk or severe neglect, but patients were not excluded because of such risks provided these risks were adequately contained in their current care setting and the primary medical responsibility for care was with the referral team. They were also excluded if they were pregnant, did not speak fluent English or had depression secondary to another primary psychiatric or organic condition. Patients were recruited from the existing caseloads of secondary care mental health teams at each site. Table 1 shows sociodemographic and clinical characteristics of the sample in the RCT at baseline, primary outcome results from baseline to 18 months, and contacts with psychiatrists and psychotherapists over 18 months (see [8] for more details). The cohort recruited was substantially more depressed, functionally impaired, and had both depression symptoms and treatment for longer than required for entry to the study. As such, they represent a group that presents challenges for treatment and management and that frequently have poor outcomes.

**Table 1:** Baseline characteristics of the main RCT sample, outcomes and care received

	TAU	SDS
	(*n* = 94)	(*n* = 93)
Age, mean (sd,)	46 (11.3)	47 (11.6)
Gender, female, n (%)	60 (64)	54 (58)
Employment status, n (%)	[*n* = 91]	[*n* = 90]
Full-time employment	22 (26)	17 (19)
Other employment^a^	11 (12)	10 (11)
Retired	10 (11)	16 (18)
Unemployed	37 (41)	36 (40)
Married or co-habiting, n (%)	50 (53)	42 (45)
Years since first diagnosis of depression mean (sd)	16.9 (11.6)	16.5 (11.1)
Depressed > 1 year, n (%)	82 (87)	80 (86)
HDRS_17_, baseline mean (sd)	23.2 (5.8)	22.0 (4.5)
6 month mean (95% CI) drop	−3.76(−5.45, −2.07)	−4.77(−6.32, − 3.22)
12 month mean (95% CI) drop	−4.99(− 7.04, − 2.94)	−7.44(− 8.98, − 5.90)
18 month mean (95% CI drop)^*^	−6.00(− 8.13, − 3.87)	−8.96(− 10.64, − 7.28)
GAF, baseline mean (sd)	47.7 (9.4)	49.3 (6.8)
6 month mean (95% CI) gain	4.61(1.51, 7.70)	5.93(3.08, 8.79)
12 month mean (95% CI) gain	5.14(1.99, 8.28)	9.26(6.31, 12.21)
18 month mean (95% CI) gain**	5.60(2.17, 9.03)	9.42(6.53, 12.31)
Median (range) number of appointments		
With psychiatrist, 0–18 months	8 (0–45)	17 (0–92)
With psychotherapist, 0–18 month	4.5 (0–49)	18 (0–67)

*HDRS17* 17 item Hamilton Depression Rating Scale

*GAF* Global Assessment of Function

*SDS significantly more effective than TAU *p* = 0.015

**SDS non-significantly more effective than TAU, *p* = 0.113

SDS = Specialist Depression Service; TAU = treatment as usual

^a^Other employment: part-time, sheltered and voluntary employment and higher education

### Participants in the qualitative study

Semi-structured interviews were conducted by MB, an experienced qualitative interviewer, with a maximum variance sample of service users from both the SDS (*n* = 12) and TAU arms (*n* = 9) at 12–18 months after baseline at the end of SDS treatment (total sample *n* = 21) (Table 2). The criteria for achieving the maximum variance sample were: study site, dropping out of follow-up, dropping out of treatment, completing treatment and completing of follow up, gender, age bands (18–30, 31–45, 46–55, over 55 years old at baseline), marital status, living on own or someone else, having a children or not, completed education at 18 or not, in work or not, time since first diagnosis, current medical comorbidity or not, other current mental comorbidity or not, moderately severe or severe at baseline, whether or not has psychotherapist at baseline, whether or not has care coordinator at baseline. Twenty one participants were selected for interview as this was the number required to check off all of these criteria. A topic guide was used to structure the interviews (Table 3). All interviews were audiotaped.

**Table 2:** Demographic and clinical characteristics of the qualitative sample

UIC	Site	Arm of the Study	Completed Treatment (6 m+)	Follow Ups Completed (up to 24 months +)	HRSD baseline	HRSD 18 months
SU15	N	SDS	Yes	Yes	21	23
SU1	N	SDS	Yes	Yes	20	11
SU24	N	SDS	Yes	Yes	24	1
SU27	N	SDS	Yes	Yes	24	6
SU7	N	SDS	Yes	Yes	27	10
SU25	N	SDS	Yes	Yes	17	5
SU11	N	TAU	N/A	Yes	16	18
SU12	N	TAU	N/A	Yes	25	24
SU17	N	TAU	N/A	Yes	16	6
SU23	N	TAU	N/A	Yes	27	19
SU2	C	SDS	Yes	Yes	25	12
SU19	C	SDS	Yes	Yes	20	18
SU16	C	SDS	Yes	Yes	22	3
SU29	C	TAU	N/A	Yes	26	22
SU30	C	TAU	N/A	Yes	19	14
SU21	D	SDS	Yes	Yes	16	8
SU4	D	SDS	Yes	Up to 12 m	19	DNC
SU8	D	SDS	Yes	Yes	24	25
SU6	D	TAU	N/A	Yes	18	29
SU18	D	TAU	N/A	Up to 18 m	19	18
SU26	D	TAU	N/A	Up to 18 m	18	20

*N* Nottingham, *C* Cambridge, *D* Derby; *SDS* Specialist Depression Service, *TAU* treatment as usual; *HDRS* 17-item Hamilton Depression Rating Scale, *DNC* Did Not Complete

Completed Treatment: Completed up to 6 m or more

Time of interview: Interview was conducted after this time point

**Table 3:** Interview Topic Guide

1. What can you tell me about how you became involved in this research?	
• Who told you about it	
• How they told you	
• Why you wanted to be part of this research	
2. Can you tell me why this research is needed?	
• What does this service offer now?	
• Do you know what the desired outcome of the research is?	
3. Do you have views on how you would like the service here to change?	
4. Do you know who is involved in this research, which doctors, nurses or other people?	
5. What are you hoping to get from your involvement with the research/service?	
6. In addition to the services you receive here, do you take any other steps to relieve your symptoms?	
7. Could involvement in this research lead you to do anything different?	
8. Have you seen changes in the service since you first became a service user?	
9. Do you know what has helped to make change happen? (give examples)	
10. Or got in the way of change happening? (give examples)	
11. Looking at the research and the efforts to improve the service, are there any things you would have liked to see done differently?	
12. Is there anything else that you would like to tell me?	

### Analysis

All interviews were transcribed by MB and NVivo 10 was used to manage the transcripts and data coding. Inductive thematic analysis using a grounded approach was adopted [27]. Initially, all transcripts were read carefully to identify sections of text relevant to service users’ experience of SDS or control arm. LT analysed the transcripts for each interview separately and generated initial codes from the relevant data using open coding. Emerging codes were tested and modified by constant comparison. Coded data were then collated into broader themes and sub-themes, which were reviewed with other members of the research team (RM, CKH, AG), before the definition and labelling of each theme were agreed upon. Quotations were selected to illustrate each theme.

## Results

Four main themes emerged from the data that related to service user experience: specific treatment components of the SDS; the individual qualities of clinicians; the collaborative team approach; and the accessibility of the SDS treatments.

### Treatment components of the SDS

Participants were able to identify specific elements of the treatment that they had received from clinicians delivering the SDS that had been particularly effective or different from their experiences of previous treatment. The sub-themes that emerged described those elements of the SDS treatments that participants had valued.

#### Management of Medication Changes

Participants who had received the SDS commented on the efficiency and clarity of any medication changes that were made during their treatment. Any changes to medication were discussed, and different options explained, so that the service user felt informed and engaged with the decision-making.

*“not only did she make suggestions but she explained kind of a route through so it is like ‘well we can try this, but if this doesn’t work then we have got other options and …. which way do you think would be best to go first?’ and so you kind of knew more where you were” SU2 (SDS).*

Furthermore, any changes made to medication through the SDS were very rapidly implemented, and avoided delays that had been commonly experienced in previous treatments.

*“if they said we want to change it, you would change it then it would happen that day” SU1 (SDS).*

#### Explaining and developing treatment strategies

Participants described the positive experience of clinicians explaining aspects of their depression, medication and therapies to them as part of their treatment within the SDS. They gained benefits from feeling more informed about and developing a more objective view of their condition.

*“there is stuff I don’t know and stuff I don’t understand and he will explain it to me, he will get a flipchart out right I will show you. This is how your brain works this is what that does if you feel like that, it triggers that and it’s wonderful, and I like just being able to understand it makes me feel a lot better….. he helps me to have some objective view of myself” SU21 (SDS).*

This understanding supported the participants to use the cognitive behavioural strategies more effectively after the trial had ended.

*“I feel I have been struggling a bit since I finished but... but then I worked out my own sheets, where I chart my mood every day …. So as soon as [my mood] starts to drop I try and be on it and do something about it.” SU25 (SDS).*

In comparison, those in the TAU arm described a cycle of routine appointments that did not engage them in their treatment and gave them no optimism for the future.

*“my current a doctor once every 3 months…. Once every 3 months. … That is no help. It is just keeping the paperwork up to speed isn’t it” SU12 (TAU).*

#### Setting realistic expectations

Participants described gaining a more realistic expectation of what their recovery might look like through their interactions with clinicians from the SDS. This largely concerned adjusting their assumptions that recovery meant being free from symptoms and no longer taking medication. Participants receiving the SDS described how they had accepted that periods of depression may still occur and that they may need to stay on some form of medication for the longer term.

*“I always assumed that I would get better and that would mean not being on tablets. … I think I spent time with [clinician], really, he’s got me used to the idea that I will probably be on something to keep me level in the long term. I think I’ve just about got used to that idea now. Whereas before, it was just completely out of the question.” SU15 (SDS).*

#### A person-centred approach

Clinicians delivering the SDS provided an individualised and person-centred approach to service users. This was characterised by asking service users about their needs and allowing them to take the lead in setting the agenda and focus for the sessions.

*“I am asked what I want to talk about at the beginning of every session, what do I think will help me, what do I want, what is making me unhappy, you know what is working for me so, …. it is different, it’s very different and a little disconcerting at first” SU21 (SDS).*

In contrast, participants in the TAU arm had not experienced this individualised, tailored approach and expressed frustration that their treatment did not address their individual needs.

*“we are not all the same, yes we might be suffering from the same symptoms but we are all different people, and we all need handling in different ways” SU30 (TAU).*

#### A holistic approach

Related to this focus on the individual person, the service users also experienced a more holistic approach from clinicians in the SDS. Participants found that the clinicians were interested in their overall well-being and quality of life, including physical health, social activity and employment, rather than just their mental health. Clinicians provided practical help in accessing physiotherapy, attending social groups and gaining benefits advice. This was in contrast to participants’ experience of previous treatments which had solely focused on their mental health. Service users also reflected that they had learnt how inter-related their mental health was with other aspects of their health and life.

*“The fact that they looked at all of my health sort of mental and physical because the two are intertwined I mean they can’t be separated ... and I have understood more clearly that that is the case since I have been on the study. That was something that was never looked at before, so all that the people before were concerned about was the mental health side of things, and I wasn’t even asked about any of the physical health or anything like that.” SU2 (SDS).*

### Individual qualities of SDS clinicians

In addition to specific elements of the treatment that they had received through the SDS, participants described the personal qualities and behaviours of individual clinicians as being important aspects of their experience of the service. A range of qualities were described by participants including: being calm and relaxed, empathetic, non-judgemental, re-assuring, positive, and having the ability to get people to open up. This last quality was particularly highlighted by participants and contrasted with their previous experience of treatments. Participants described developing a connection with the clinicians, allowing them to open up and trust their clinicians which they believed had helped with their engagement with the treatment.

*“I find this a lot better, … I have been able to open up more... probably share more things than I ever have shared any before ... I don’t know whether it’s because of this particular [clinician] the way she is, has... worked with me and allowed me to open up …. I found I have been able to talk openly and honestly with her.” SU8 (SDS).*

The importance of making a positive and trusting connection with a clinician was highlighted by one participant from the TAU arm of the study who had not experienced a positive working relationship.

*“I think it was of limited use because I didn’t get on with the therapist…. she wasn’t somebody I could really open up to, or talk to, I guess you know I didn’t trust her …. just not somebody I felt comfortable [with]… because I didn’t feel that she got me or understood me or whatever so, you know that puts a barrier between you kind of opening up.” SU17 (TAU).*

In contrast, a few participants in the TAU arm did experience a more positive relationship with their clinicians, and found this beneficial to their treatment.

*“she was the only one really that I think understood... I really was grateful because the understanding has never been there before if you know what I mean” SU23 (TAU).*

### A collaborative team context

Service users who had experienced the SDS were able to articulate the differences in their treatment due to the collaborative team approach. These focused on two main aspects: improved communication between healthcare professionals, and continuity amongst team members.

#### Communication between healthcare professionals

It was obvious to service users that the clinicians involved in the treatment had been sharing information and discussing aspects of their treatment. The knowledge that the clinical team were all talking to each other about the service users’ treatment was identified as significantly different from previous experiences, and gave service users confidence in the approach to their treatment, and that all clinicians involved were fully informed about other aspects of their treatment.

*“They seem to work so well as a team, … I can speak to someone like [therapist] and I will see [psychiatrist] two or three weeks later and she will speak to me about what [therapist] said, so I know it gets passed on, I know they are all talking to each other.” SU16 (SDS).*

In contrast, participants in the TAU arm were not confident about the exchange of information between different clinicians that may be involved in their care.

*“I have only got my psychiatrist really to talk to, and he was showing me ways by getting in touch with a separate organisation to, but then they won’t be talking to him to kind of you know [about me]” SU29 (TAU).*

#### Continuity of team members

Having clinical team members that were consistent and unchanged throughout the study was also experienced as something quite different from previous treatments and the TAU arm. This continuity was described as being beneficial to developing trust and confidence in the clinicians, which in turn benefitted their engagement with treatment.

*“I think the most important factors are the continuation of the same people, and the distinction between their different roles… It give me confidence in them.” SU2 (SDS).*

In contrast, the TAU participants described the impact of seeing different clinicians during treatments, both during the study and through prior experience. Many participants from both arms of the study described their previous experiences of constantly changing psychiatrists and the frustrations of having to retell their experiences from the beginning on multiple occasions. In addition, the uncertainty and concern that was experienced due to changing clinicians was felt to be particularly damaging for people with depression.

*“change for me isn’t a good thing because I have to reset all my, or rearrange all my parameters … what makes me feel safe and stuff, so seeing somebody new … in general was a problem” SU30 (TAU).*

### Accessibility to SDS

Participants who had experienced the SDS described the benefits of an easily and frequently accessed service.

#### Access outside appointment times

Participants felt reassured that they could access the service to make additional appointments to those already made if they needed to. In reality, they rarely made additional appointments but the knowledge that this was available appears sufficient to convey some benefit to the service users.

*“knowing that if things were bad, I could phone her up and get an urgent appointment with her, was more valuable than actually doing it to be honest. … I can’t stress enough how important that is, really important.” SU24 (SDS).*

This contrasted sharply with the experience of participants in the TAU arm who described the challenges of getting earlier appointments or talking to someone between appointments, and negative impact of having a service unresponsive to the needs of service users at critical times.

*“It is like trying to get into Fort Knox, to try and maybe get an earlier appointment. I have rung the secretary and said ‘I really could do with...’ By the time I get one it’s my appointment time anyway.” SU8 (TAU).*

#### Flexible locations

A number of participants described the benefits of being able to have consultations take place in their homes. This flexibility provided both reassurance and a sense of safety to the service users who had requested this. But one participant also thought it gave the clinician some additional insights into the service user’s state of mental health.

*“It’s been really, really good that she has come to me most of the time at home, because that’s, that is really significant actually…. I would think it helps [the clinician]... because she can see at any given time how well I am kind of looking after things,... she has definitely seen it in various states, but also just for her to know the environment in which I am in.” SU2 (SDS).*

#### Frequent consultations

Participants reported that the frequent and ongoing consultations in the SDS was of benefit to them. They felt that their treatment was very proactive with more time invested by clinicians. This was particularly important in relation to their application of CBT techniques and skills learnt in consultations, and the need for reminders and reinforcement of the techniques learnt.

*“There were a couple of times where I was on holiday or [the clinician] was on holiday and you know it was two weeks [between appointments] and it just gives you that much time to slip back a bit. Whereas if you have got a week, you can keep it in your head and you can work on whatever the ideas that you are working on. And then remember it and feedback, quite effectively.” SU24 (SDS).*

By the end of the trial some service users reported a decline when the frequent consultations had stopped, again pointing to the benefits of having regular reminders of the CBT techniques.

*“I’m almost a year out of the project now and I can feel I’m slowly slipping back down again. But there’s more regular times on the [trial] and that year afterwards. It did away with all of that and I did feel there was some hope.” SU15 (SDS).*

In contrast, service users in the TAU described how the infrequent sessions and time-limited nature of the service prevented them making progress, largely through the additional time taken to build an effective therapeutic relationship.

*“you are talking about 4 or 5 almost 6 sessions to really start trusting somebody because each time they chip away at you and you, you let a bit out and you are still guarded and yes, so you are talking what 3 months say, you know before you are really opening up completely. And obviously you are not going to get anywhere unless you open up to these people, you are not going to get any better” SU29 (TAU).*

#### Gradual pace of treatment

Participants receiving the SDS frequently reported that the 12 month duration of treatment allowed a better pace to be established. Participants didn’t feel rushed which in turn led to better engagement, and more established recovery allowing service users to recover better from periods of depression.

*“you can do it so rigorously when it’s once a week for a whole year it’s really, it just becomes part of like the foundation, the missing bits of foundation. And then it’s I don’t know it’s just there for you to fall back on” SU25 SDS.*

Having time to get service users to the point at which they can start to fully engage with the treatment and the CBT techniques was seen as one of the benefits of a longer treatment cycle. A participants receiving the Treatment as Usual arm described this in relation to time-limited therapy sessions.

*“you get your 20 sessions or your 10 sessions or whatever, [but] what if you are in a state where it takes 6 or 7 sessions to get to the point where you can intellectually connect with the process and really do it properly…. And it doesn’t have time to take effect and yes it gets you out of the, being in a pit of misery but it doesn’t stop you going back into it the next time something horrendous happens.” SU24 TAU.*

#### Challenge of returning to TAU

Some participants who had received the SDS found the transition back to the usual frequency of appointments a difficult process. Moving from the enhanced accessibility and frequency of their treatment in the SDS arm of the trial to the more infrequent sessions experienced in usual care also meant that they were subject to the difficulties of cancelled appointments and changing clinicians.

*“Well I mean that was appalling really in the sense that you know you have been seeing somebody regularly and trustworthingly…, you then got referred back to your GP basically and the mental health services ….[and that appointment was cancelled] so it was 4, nearly 4 or 5 months or something in that order before I next saw a... mental health professional” SU1 (SDS).*

Participants also missed the frequent contact with clinicians and described how they need to be more proactive in their own use of the CBT techniques.

*“CBT teaches you to almost not have to go tell people things doesn’t it, it’s like you are your own sort of mentor aren’t you but, I think there is also part of me needs to just talk things over with somebody from time to time I think… But, I just lost my objectivity a bit, … So maybe if I just had a chat with someone at 3 months perhaps” SU25 (SDS).*

Further difficulties were experienced by participants due to lack of clear communication about the end of the trial and the transition back to usual care.

*“I didn’t realise my last session with him was my last session… And he came in and said, last one. And I just burst into tears. But that was, I was scared, I thought I wasn’t going to have that one last throw of the dice, if you like.*” *SU15 (SDS).*

However, the longer-term impact of the SDS was reported by some of the participants.

*But there’s more regular times on the [trial] and that year afterwards, ….. It did away with all of that and I did feel there was some hope. SU15 (SDS).*

## Discussion

The service user experience of the SDS was very positive, and participants were able to articulate elements of the service which differed from their previous experiences of treatment, and why they were beneficial to them. Participants felt informed and engaged in the different elements of their treatment, both in relation to their CBT and their medication. In the SDS, participants felt that different options were explained and set out, and that they had some control in setting the agenda and having their individual needs addressed. This included having clinicians help with other aspects of their well-being and quality of life. Having realistic expectations about what their recovery might look like was another important element of the SDS that participants experienced. This frequently involved accepting that it might be necessary to stay on some form of medication and that depression symptoms may reoccur. Many of these elements align themselves with recovery-focused practices which aim to achieve service user defined goals and outcomes to build a meaningful and satisfying life that is not necessarily symptom free [28].

The personal qualities of clinicians were also highlighted as an important element of effective treatment, particularly in developing trust and encouraging the participants to open up to the clinicians. This wasn’t unique to the SDS arm, and similar qualities were experienced by people in the TAU arm too. This finding highlights the benefits of developing positive working relationships between service users and mental health professionals, as has been identified in other studies [22, 29].

Aspects of the SDS collaborative team working were also identified by participants as a beneficial feature of their treatment that they had not experienced before. Participants gained confidence in the knowledge that the different clinicians involved in their treatment were communicating effectively with each other, and informed about the different aspects of treatment. The continuity of team members throughout treatment further enhanced this communication and also supported the development of trust between clinicians and service users, resulting in better engagement. This was sharply contrasted by the participants’ previous experience of frequent changes to clinicians and the negative impact this had on their treatment.

Finally, participants described the benefits they experienced from the ease and frequency of access to the SDS. Although they rarely accessed the service outside of their booked appointments, knowing that it was possible to get earlier appointments reassured the service users. Having appointments at home was also perceived to be beneficial for a number of participants. For the CBT part of the service, the frequency of consultations and total length of treatment were both highlighted by participants as important factors that contrasted with their previous experience of treatment. Frequent CBT sessions helped participants to reinforce the techniques used, and avoid slipping back into their usual ways of thinking. Similarly, having a sufficient number of CBT sessions was repeatedly stressed as being important to allow service users to fully engage with the therapy, develop trusting relationship with the clinician and establish the techniques to support their prolonged recovery. The importance to service users of the appropriate timing of treatment which coincides with their own readiness has been found in other studies [22, 30].

Although the frequency of consultations experienced by participants in the SDS arm of the trial was perceived by them as one of the benefits of this approach, this also resulted in some challenges for participants once they returned to treatment as usual when the trial had finished. The transition into usual care was difficult for some participants to adjust to. However, there was evidence from the service user interviews in the SDS arm of changes in attitudes to treatment and coping strategies indicating lasting improvement in coping with this long-lasting and recurrent condition.

### Strengths and limitations

Qualitative methods were used in this study to explore and understand the experiences of service users with long experience of mental health services to provide additional insights into the mechanisms of action of the SDS on depression symptoms versus TAU. Important aspects of care can be identified in a complex intervention such as a service intervention that are difficult to capture otherwise. Most RCTs of single pharmacological or psychological treatment interventions can explore the process of care delivery through simple methods such as prescribed medication and therapist competency but care in services covers many interdependent therapeutic approaches delivered by many different staff.

Whilst this approach enables greater depth of understanding of service user experience, the generalisability of findings is limited by the nature of the sample, which is both small and self-selecting. We did not have interviews with patients who dropped out early from the SDS and may have had much more negative experiences of such treatment. The contrast between the SDS and TAU may be more or less marked if usual care in specialist general mental health services has fewer or more features in common with the SDS care outlined here. Furthermore service user experience may not reflect accurately important aspects of care that is technical in nature. It also did not provide insight into the non-significant effects of SDS on functional outcomes as opposed to the effectiveness on depression symptoms.

### Implications for practice

Service users highlighted the benefits of features of the SDS service which were different from their previous experiences of treatment and those in the control arm. Participants positive experience of the SDS centred around the ability to develop trusting relationships with therapists working in a stable and collaborative team, the frequency and accessibility of sessions and the ability to top-up and reinforce the techniques learnt over a longer period. In addition, having a sufficiently long treatment period to foster the development of trust and positive relationships is important; service users should not feel rushed. Participants spoke about the importance of being able to engage with the therapists which required building up trust and connections. The need for a gradual pace and length of treatment was reflected in the outcome measures of the RCT where there was no difference between the arms in any outcome measure until 6 months and significance on the primary HDRS outcome measure was not reached until 18 months [8]. Time-limited CBT sessions will not achieve these factors that were widely considered to be most beneficial. These findings concur with other studies which have demonstrated that short-term therapies are insufficient in leading to recovery. A meta-analysis of six trials comparing short-form psychodynamic psychotherapy with CBT for depression found that 16–20 sessions of either therapy was insufficient to achieve a long-term remission for patients [15]. A more recent trial [16] also found that more than 16 sessions of either CBT or psychodynamic supportive therapy were needed to achieve remission in patients attending outpatients’ clinics. Together, these findings suggest the future design of services for people with recurrent depression should aim to foster a longer-term and collaborative approach to building therapeutic relationships.

## Conclusions

This study provided in-depth qualitative data about service users’ experience of the SDS, how it differed from their previous experiences of treatment, and why this was beneficial to them. The findings highlight the importance of some of the specific treatment components of the SDS model, such as the careful management of medication change, taking time to explain treatment strategies, setting realistic expectations in relation to recovery, and using a person-centred and holistic approach. In addition, the individual qualities of clinicians were highlighted as important in developing trust with service users, which promoted better engagement with clinicians. The collaborative nature of the SDS team provided a context of effective communication and continuity within the team, which further promoted confidence amongst service users. Finally, frequent consultations and ease of access supported the experience of service users, allowing them to engage in therapy at their own pace. However, the frequency of consultations also resulted in some challenges for participants once they returned to treatment as usual when the trial had finished. The transition into usual care was difficult for some participants to adjust to. However, there was evidence from the service user interviews in the SDS arm of changes in attitudes to treatment and coping strategies indicating lasting improvement in coping with this long-lasting and recurrent condition.

## Abbreviations

CBT: Cognitive Behavioural Therapy
GAF: Global Assessment of Functioning
GP: General Practitioner
HRSD: Hamilton Rating Scale for Depression
NICE: National Institute for Health and Care Excellence
RCT: Randomised Controlled Trial
SDS: Specialist Depression Service
SU: Service User
TAU: Treatment as usual
